# Effects of inclusion of purple prairie clover (*Dalea purpurea* Vent.) with native cool-season grasses on *in vitro* fermentation and *in situ* digestibility of mixed forages

**DOI:** 10.1186/s40104-019-0418-6

**Published:** 2020-02-14

**Authors:** Kai Peng, Gemma L. Gresham, Tim A. McAllister, Zhongjun Xu, Alan Iwaasa, Mike Schellenberg, Alex V. Chaves, Yuxi Wang

**Affiliations:** 1 Key Laboratory of Animal Nutrition and Feed Science (South China) of Ministry of Agriculture, Guangdong Key Laboratory of Animal Breeding and Nutrition, Institute of Animal Science, Guangdong Academy of Agricultural Science, Guangzhou, 510640 People’s Republic of China; 2 Agriculture and Agri-Food Canada, Lethbridge Research and Development Centre, Lethbridge, AB T1J 4B1 Canada; 3 School of Life and Environmental Sciences, The University of Sydney, Sydney, NSW 2006 Australia; 4 Agriculture and Agri-Food Canada, Swift Current Research and Development Centre, Swift Current, SK S9H 3X2 Canada

**Keywords:** Condensed tannins, Cool-season grass, *In vitro* fermentation, *In situ *digestibility, Purple prairie clover

## Abstract

**Background:**

Incorporation of legume species into native North American pastures is considered an effective method to increase native pasture productivity and improve the nutritive value of forage. This study evaluated the effects of inclusion of purple prairie clover (PPC, *Dalea purpurea* Vent.), a native legume forage, with native cool-season grasses on the *in vitro* fermentation and *in situ* digestibility of mixed forages.

**Methods:**

Whole plant PPC and mixtures of cool-season grasses were harvested when the PPC reached the vegetative (VEG), full flower (FL) and seedpod (SP) stages, and were combined in ratios (DM basis) of 0:100, 25:75, 50:50, 75:25 and 100:0 at each maturity. *In vitro* ruminal incubations using these mixtures were conducted for 48 h to determine gas production (GP), *in vitro* DM disappearance (IVDMD), total volatile fatty acids (VFA) and ammonia-N production. Mixtures of forages harvested when the PPC reached the FL stage and 50:50 mixture of forages harvested at VEG, FL and SP stages were incubated in the rumen of three heifers for 0, 2, 6, 12, 24, 48, 72 and 96 h to determine *in situ* degradabilities of DM, neutral detergent fibre (aNDF) and crude protein (CP).

**Results:**

Contents of aNDF and ADF increased (*P* < 0.01), while CP decreased (*P* < 0.001) as PPC matured. Concentrations of extractable condensed tannins in PPC ranked as FL > VEG > SP (*P* < 0.05). Regardless of PPC proportions in the mixture, GP decreased (*P* < 0.05) with increasing PPC maturity. Increasing PPC proportions linearly increased (*P* < 0.001) GP, IVDMD and total VFA at VEG, but linearly decreased (*P* < 0.001) them at SP. Irrespective of PPC maturity, ammonia-N production linearly increased (*P* < 0.01) with increasing proportions of PPC and the concentration was higher (*P* < 0.05) at VEG than at FL and SP stages. Increasing proportion of PPC at either maturity linearly increased (*P* < 0.001) molar percentage of acetate (A) and branched-chain VFA, but linearly decreased (*P* < 0.001) molar percentage of propionate (P), resulting in a linearly increase (*P* < 0.001) in the A:P ratio. Increasing FL PPC in the mixture linearly and quadratically (*P* < 0.01) increased *a* (soluble fraction), but linearly and quadratically decreased (*P* < 0.01) *b* (potentially degradable fraction) for DM and aNDF, resulting in linear (*P* < 0.05) and quadratic (*P* < 0.01) increases in DM and aNDF maximum potential degradabilities (*a* + *b*). Effective degradabilities of DM and aNDF were also linearly and quadratically increased (*P* < 0.05), and CP was quadratically increased (*P* < 0.05) with increasing FL PPC, with the greatest effective degradability being observed with ratios between 50:50 and 75:25. Ruminal maximum potential degradabilities of DM and aNDF decreased (*P* < 0.001) as the forage matured. Effective degradability of DM ranked as VEG > FL > SP (*P* < 0.001), whereas the effective degradability of aNDF was similar between VEG and FL and both were greater (*P* < 0.01) than SP.

**Conclusions:**

Inclusion of vegetative PPC in a mixed forage diet resulted in the greatest digestibility and incorporation of PPC before seedpod stage with native grasses had a positive effect on ruminal fermentation. Effects of PPC on ruminal digestion depend on both the stage of maturity and its proportion in mixed legume-grass pastures. Pastures containing 50% of PPC in full flower stage would likely provide the greatest quality diet to grazing ruminants subject to potential animal selectivity.

## Background

Grasses in native pasture are usually the principal forage source in the prairie region of North America during late season grazing. However, nutritive value of grasses rapidly declines in the latter half of the grazing season [1]. Inclusion of native legume species in rehabilitated native prairie pasture in North America is considered an effective method to increase pasture productivity and quality while increasing the protein concentration of the forage [2, 3]. Rehabilitation of native pasture is the process of re-establishing the structure, function, and integrity of native ecosystems and their habitats. The agronomic and nutritional advantages of inclusion of legumes in grass-based pasture systems have been well documented [4–7]. These include increased pasture productivity, herbage nutritive value and resource efficiency through the symbiotic nitrogen fixation and thereby improve animal performance. As a result, this practice can extend the grazing season and reduce the cost of beef production on native pasture.

Purple prairie clover (PPC, *Dalea purpurea* Vent.) is a perennial native legume that is well adapted to the North American prairies and has higher palatability and digestibility than other native legumes such as false indigo (*Amorpha fruticosa *L.), blue wild indigo (*Baptisia australis* (L.) R. Br.) and wild senna (*Senna hebecarpa* (Fernald) Irwin & Barneby) [8]. The PPC generally blooms July through September and has the ability to extend and improve the sward forage quality during the entire grazing season. These properties could enable PPC to be the most desirable native legume for the remediation of prairie native grass pastures [9]. In addition, PPC contains high concentration of condensed tannins (CT) that possess antimicrobial, anti-parasital, antioxidant, anti-bloat properties and modulate the immune system of animals [10]. Our previous studies showed that PPC CT up to 82 g/kg DM had varying impact on ruminal feed digestion and animal growth performance depending on the conservation method of PPC and its proportion in the diet [11–13]. Therefore, defining the optimal levels of PPC in consumed mixed forages is needed to target the most desirable density of PPC in mixed native grass pastures. Although the *in vitro* ruminal digestion of PPC and PPC-grass mixtures have been evaluated [3, 11], little information is available on the impact of PPC on ruminal digestion of PPC-grass mixtures at varying ratios and stages of maturity. This information is needed to estimate the optimal density of PPC in rehabilitated native pasture.

The objective of this study was to assess the impact of mixing different levels of PPC at varying stages of maturity with native cool-season grasses on the *in vitro* ruminal fermentation and *in situ* nutrient degradation.

## Materials and methods

### Forage preparation

Whole plant PPC (AC Lamour) and a mixture of cool-season grasses including western wheatgrass (*Pascopyrum smithii* (Rydb.), WR Poole), northern wheatgrass (*Agropyron cristatum*, Critana), green wheatgrass (*Elymus hoffmannii* Jensen & Asay, AC Mallard), little bluestem (*Schizachyrium scoparium*, Badlands), blue grama (*Bouteloua gracilis*, Bad River), awned wheatgrass (*Agropyron cristatum*, AC Pintail), Canada wildrye (*Elymus canadensis* L., Mandan), needle-and-thread grass (*Hesperostipa comate*, AC Sharptail) were collected from three rehabilitated native pastures that were seeded in 2011. Fertilizer (11–51-00) was used as a seed carrier during seeding to prevent seed bridging. The ratio of seed mix to fertilizer was 1:1 and was seeded at a rate of approximately 9 kg/ha. The PPC seed was 2% of the seed mixture. The pastures were located at the Swift Current Research and Development Centre (SK, Canada; latitude N50°17′, longitude W107°41′, 825 elevation) on a Swinton Loam soil (Orthic Brown Chernozem) [11]. Samples of both grasses and PPC were collected with a pair of scissors about 2.0 cm above ground level from three locations in each pasture when the PPC reached the vegetative (VEG; June in 2015), full flower (FL; July in 2015) and seedpod (SP; August in 2015) stages of maturity of PPC. Upon collection, PPC was manually separated from mixed grasses and each was composited by pasture and maturity and freeze-dried [12]. Dried samples were ground through a 1.0-mm screen, with PPC and the grass mixture from each pasture at each maturity were combined in ratios (PPC: grasses) of 0:100, 25:75, 50:50, 75:25 and 100:0.

### Determination of *in vitro* ruminal fermentation of forage mixture

Approximately 0.5 g DM of each forage mixture was weighed into acetone-washed, pre-weighted F57 filter bags (pore size of 25 μm; ANKOM Technology Corp.) [12]. Bags were sealed and placed in 125-mL serum vials in preparation for *in vitro* ruminal batch culture fermentation.

Inoculum was prepared on the day of the incubation using fresh rumen fluid that was collected 2 h after the morning feeding and combined in equal volumes from three ruminally cannulated Angus heifers (480 ± 5.5 kg, 32 months). The heifers were fed (DM basis) a forage diet containing 50% alfalfa hay, 35% barley silage, 12% dry-rolled barley and 3% of a vitamin and mineral supplement as per the National Research Council [14] recommendations. All heifers were fed at 08:00 h and provided *ad libitum* access to feed and water and were cared for in accordance with standards of Canadian Council on Animal Care [15]. Rumen fluid collected from five locations within the rumen was strained through 4 layers of cheesecloth and immediately transported in an anaerobic and pre-warmed container to the laboratory. Rumen fluid was then combined (1:3, v/v) with pre-warmed mineral buffer (39 °C) [16] to generate the inoculum.

Vials containing substrate were warmed to 39 °C and flushed with O_2_-free CO_2_ prior to the addition of 60 mL of inoculum. Vials were immediately sealed and affixed to a rotary shaker platform (160 r/min) housed in a 39 °C incubator (Forma Scientific reach-in incubator, Model # 39419–1, 120 V, 60 Hz). Triplicate vials containing inoculum without substrate were also incubated to serve as blank controls. Vials for 0 h incubation were placed on ice immediately following addition of inoculum.

Headspace gas production (GP) was measured in the vials at 3, 6, 9, 12, 24 and 48 h post-inoculation by inserting a 23-gauge (0.6 mm) needle attached to a pressure transducer (model 15078–193; Fisher Scientific, Pittsburgh, PA, USA) connected to a visual display device (Data Track, Christchurch, UK). The recorded cumulative gas pressure, corrected for the gas released from the blanks, was converted to volumes (mL) using the equation of Mauricio et al. [17]:

GP = 0.18 + 3.697P_t_ + 0.0824P_t_^2^where GP is gas production, mL; P_t_ is pressure transducer reading value, psi.

Fermentation vials were removed from the incubator after 48 h of incubation and placed in ice-water. The bags were removed from vials, manually washed under running tap water until the stream was clear and dried in an oven at 55 °C for 48 h. Bags were used to estimate *in vitro* dry matter disappearance (IVDMD) by subtracting the loss of DM from the bags from the initial DM incubated. The liquid fraction was processed immediately for determinations of ammonia-N and volatile fatty acids (VFA) as described by Wang et al. [16]. Two runs of each incubation with six replicates for each treatment per run were conducted.

### Determination of ruminal degradability of the forage mixtures

Whole plant PPC and grasses harvested at FL of PPC as described above were combined in ratios of 0:100, 25:75, 50:50, 75:25 and 100:0 (PPC: grasses) to evaluate the effect of PPC on ruminal degradability of the forage mixtures. In addition, PPC and grasses harvested at each maturity of PPC (VEG, FL and SP) were combined at a ratio (DM basis) of 50:50 to determine the effect of maturity on the degradability of the mixture. For these determinations, freeze-dried PPC and grasses were ground to pass through a 4.0 mm screen before mixing and the same three heifers used as rumen fluid donors for the *in vitro* incubation were used in the *in situ* experiment.

The procedure for incubation of the nylon bags and subsequent determinations of DM, neutral detergent fibre (aNDF) and CP disappearances were the same as described by Huang et al. [12]. Mixed forage samples were weighed (5 g/bag) into nylon bags (10 cm × 20 cm, 50 μm pore size, ANKOM Technology, Macedon, NY, USA). Duplicate bags containing respective substrates were incubated in the rumen of each heifer for 2, 6, 12, 24, 48, 72 and 96 h. Nylon bags were placed into large mesh bags (20 cm × 30 cm) and soaked in warm water (39 °C) for 10 min prior to placement in the rumen. The nylon bags in mesh bags were inserted into the rumen in reverse order of incubation time so that all bags were removed simultaneously after incubation. The removed bags from the rumen were immediately rinsed under cold running tap water till the rinse water was clear and subsequently washed in a washing machine for 2 min without detergent or the use of the spin cycle. The 0 h bags were not incubated in the rumen, but were washed using the same protocol. All bags with residue were subsequently dried at 55 °C for 48 h and weighed to determine DM disappearance. Residues from duplicate bags of each sample incubated in the same heifer were pooled and ground to pass through a 1 mm screen for determinations of aNDF and CP disappearance [18].

### Laboratory analysis

Dry matter was determined by drying samples at 105 °C for 16 h in a forced-air oven (AOAC, # 930.15) [19] and organic matter (OM) was determined by ashing in a muffle furnace (AOAC, # 943.01) [19]. The samples were ball ground in a planetary micro mill (Retsch Inc., Newtown, PA, USA) and analyzed for total N estimation by flash combustion analysis using a NA1500 nitrogen analyzer (Carlo Erba Instruments, MI, Italy). Neutral detergent fibre and acid detergent fibre (ADF) were performed using an Ankom 200 system (Ankom Technology Corp., Fairport, NY, USA), with addition of sodium sulfite and alpha-amylase for aNDF but without for ADF analysis as described by McGinn et al. [20], and the residual ash was included in the aNDF calculation. The concentrations of extractable CT (ECT) of forage samples were determined using method described by Terrill et al. [21] with purified PPC CT used as a standard [22].

### Calculation and statistical analysis

*In situ* DM, aNDF and CP disappearances were determined as the difference in substrate weight before and after ruminal incubation. The kinetics of *in situ* DM, aNDF and CP disappearance were estimated using a non-linear regression procedure of SAS (SAS Institute Inc., Cary, NC, USA) using the equation described by McDonald [23]:

*P* = *a* + *b* (1 − e^−*c* (t − *L*)^)where *P* = ruminal disappearance at time *t* (%), *a* = the rapidly soluble degradable fraction (%), *b* = the slowly or potentially degradable fraction (%), *a* + *b* = the maximum potential degradability, *c* = the rate at which *b* is degraded (%/h), *t* = time (h) incubation in the rumen, and *L* = lag time (h).

Effective degradabilities (ED) of DM, aNDF and CP were estimated using the equation described by Orskov and McDonald [24]:

ED = *a* + [*bc*/(*c* + *k*)] e^−(c + *k*) *L*^with *a*, *b*, *c* and *L* as described above and *k* = the ruminal outflow rate (%/h), which was set at 0.02 for aNDF and 0.05 for both DM and CP [25]. The constants *a*, *b*, *c* and *L* for each animal were calculated using nonlinear regression procedures of SAS [26]. The degradability of CP for the mixture of 0:100 (PPC: grasses) could not be estimated due to the very low N content in these samples and thereby was excluded from the final analysis.

All data were analyzed using the MIXED procedure of SAS. Chemical compositional data were analyzed using one-way ANOVA with maturity as fixed effect and forage pasture as a random factor. Data from the *in vitro* and *in situ* studies were analyzed by completely randomized design model. Forage mixture ratio, PPC maturity and their interaction were the fixed effects and run was treated as a random factor in the analysis of *in vitro* data, whereas either forage mixture ratio or PPC maturity was considered as fixed effects and cow was treated as a random factor in analysis of *in situ* experiment data. Disappearances of DM, aNDF and CP were also analyzed at each incubation time. Orthogonal polynomial contrasts were used to determine linear and quadratic responses to the levels of PPC in the forage mixtures. The parameters calculated from the *in situ* DM, aNDF and CP disappearance data, were analyzed using MIXED model procedure of SAS using the following model:

*y*_*ij*_ = *μ* + *α*_*i*_ + *β*_*j*_ + *ε*_*ij*_where *y*_*ij*_ is the parameter, *μ* is the overall mean, *α*_*i*_ is the effect of heifer (1-3), *β*_*j*_ is the effect of treatment, and *ε*_*ij*_ is the residual error.

Differences between treatments means was determined by the PDIFF option of LSMEANS in SAS and declared significant at *P* < 0.05.

## Results

### Chemical characteristics of PPC and cool-season grasses at different maturities

In general, PPC was numerically lower in aNDF and ADF, but higher in CP than the grasses (Table 1). Contents of aNDF and ADF increased (*P* < 0.01) but CP decreased (*P* < 0.001) with advancing maturity of PPC. In contrast, these changes over the same sampling period were not as obvious for cool-season grasses, likely because these grasses were cool-season grasses that had already reached physiological maturity. Concentration of ECT in PPC was highest (*P* < 0.01) at FL, followed by VEG and SP, respectively (*P* < 0.05). Condensed tannins were not detected in any of the mixed grass samples.

**Table 1:** Chemical composition (g/kg DM) of purple prairie clover (PPC; *Dalea purpurea* Vent.) and cool-season native grasses that were harvested when PPC reached vegetative (VEG), full flower (FL) and seedpod (SP) stages

Item^e^	PPC			SEM	*P*-value	Grass			SEM^d^	*P*-value
	VEG	FL	SP			VEG	FL	SP		
OM	931.9	939.3	929.6	4.70	0.421	926.9^b^	928.5^b^	939.5^a^	1.74	0.026
aNDF	438.3^b^	526.1^a^	547.8^a^	5.64	0.002	738.5	748.7	757.4	7.27	0.323
ADF	349.8^c^	497.1^b^	534.0^a^	6.89	< 0.001	507.2	516.8	509.6	2.80	0.067
CP	137.8^a^	99.7^b^	83.4^c^	0.75	< 0.001	37.5^a^	29.4^b^	35.1^a^	0.44	0.009
ECT	65.7^b^	87.2^a^	50.4^c^	1.67	< 0.001	ND	ND	ND	–	–

^a,b,c^Means with different superscript letters in the same row differ (*P* < 0.05)

^d^*SEM* standard error of the means (*n* = 4)

^e^*OM* organic matter, aNDF neutral detergent fibre (the residual ash was included in the aNDF calculation), *ADF* acid detergent fibre, *CP* crude protein (N × 6.25), *ECT* extractable condensed tannins, *ND* not detectable

### *In vitro* ruminal fermentation characteristics of PPC and grass mixtures at different maturities

**Fig. 1:**
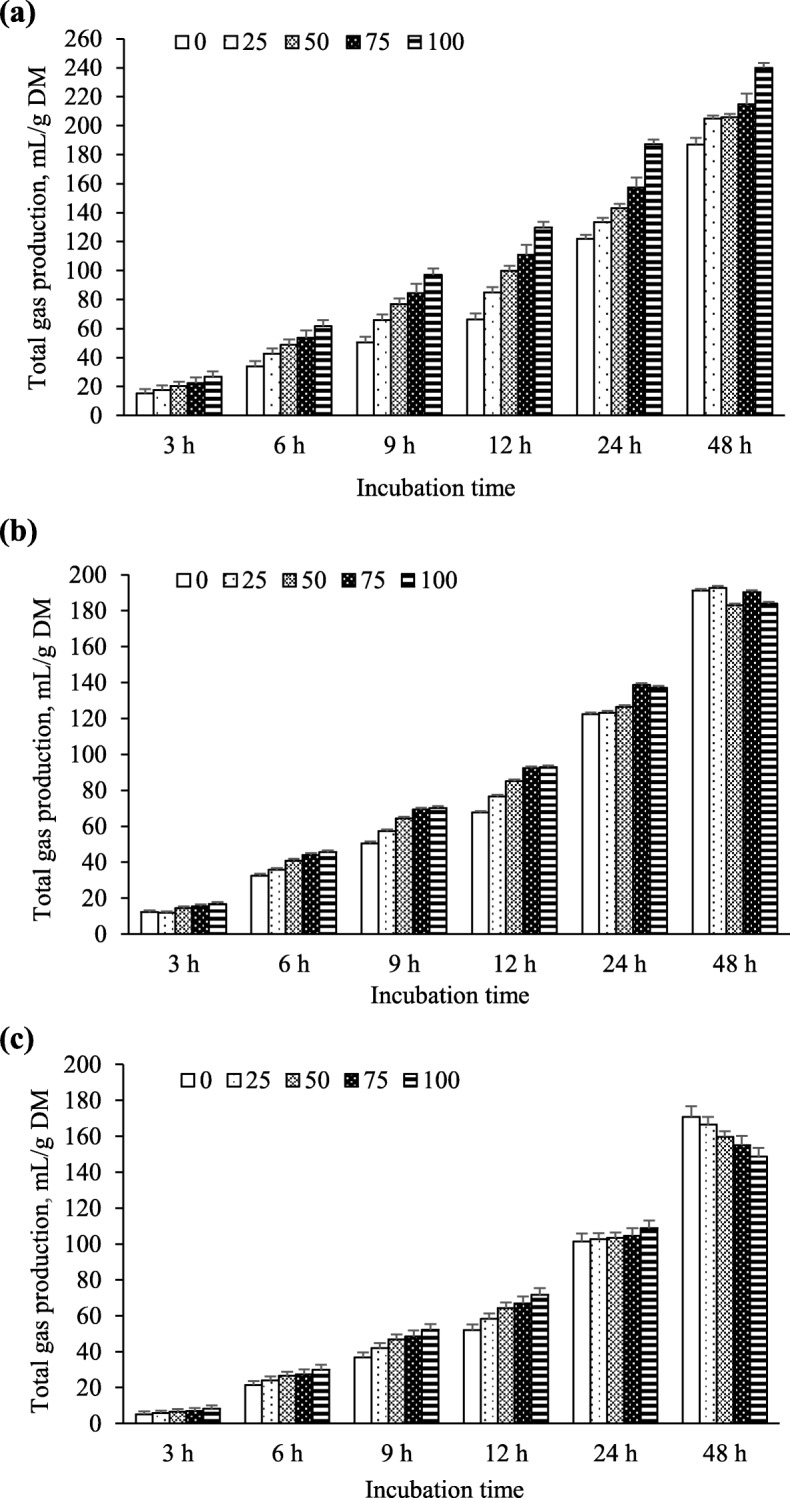
Total gas production during 48-h *in vitro* ruminal incubation of different ratios of purple prairie clover (PPC; *Dalea purpurea* Vent.) and cool-season grasses that were harvested when PPC reached the vegetative (**a**), full flower (**b**) and seedpod (**c**) stages

Fermentation of the PPC-grass mixtures differed with changes in the amount of PPC in the mixtures as well as maturity (Tables 2 and 3, Fig. 1). With increasing concentrations of PPC, IVDMD linearly increased (*P* < 0.001) at VEG, but linearly decreased (*P* < 0.001) at SP (Table 2). Dry matter disappearance was also quadratically increased (*P <* 0.05) as the VEG PPC increasing in the mixture. However, these differences were not observed for GP and total VFA when forages were at the FL stage. When plants were at the VEG stage, GP linearly increased (*P* < 0.01) over the 48-h incubation with increasing PPC in the mixture (Fig. 1a). However, this linear increase was observed only during the early periods (i.e., 3, 6, 9 and 12 h) of incubation at the FL and SP stages (Fig. 1b, c).

**Table 2:** *In vitro* dry matter disappearance (IVDMD, mg/g DM) and ammonia-N accumulation (mmol/L) after 48-h *in vitro* ruminal fermentation of purple prairie clover (PPC; *Dalea purpurea* Vent.) and cool-season native grasses that were harvested when PPC reached the vegetative (VEG), full flower (FL) and seedpod (SP) stages and combined at different ratios

Maturity (M)	PPC percentage (PP)					SEM^e^	*P* values^f^				
	0	25	50	75	100		M	PP	M × PP	L	Q
	IVDMD										
VEG	564^d^	604^c^	590^cd^	635^b^	705^a^	35.9	< 0.001	< 0.001	< 0.001	< 0.001	0.015
FL	512^a^	509^a^	463^b^	471^b^	469^b^					0.001	0.161
SP	571^a^	561^a^	507^b^	475^c^	440^d^					< 0.001	0.728
	Ammonia-N										
VEG	5.43^e^	6.00^d^	6.72^c^	7.23^b^	8.00^a^	0.293	0.049	< 0.001	0.731	< 0.001	0.772
FL	4.74^c^	5.31b^c^	5.74^b^	6.59^a^	6.87^a^					< 0.001	0.863
SP	4.87^c^	5.53^b^	5.92^b^	6.49^a^	6.71^a^					< 0.001	0.436

^a,b,c,d^Means with different superscript letters in the same row differ (*P* < 0.05)

^e^*SEM* standard error of the means

^f^*L* linear, *Q* quadratic, These are linear and quadratic contrasts across PPC percentages within PPC maturity

**Table 3:** Total volatile fatty acids (VFA) concentrations and individual VFA profiles after 48 h of *in vitro* ruminal fermentation of purple prairie clover (PPC; *Dalea purpurea* Vent.) and cool-season native grasses that were harvested when PPC reached the vegetative (VEG), full flower (FL) and seedpod (SP) stages and combined at different ratios

Maturity (M)	PPC percentage (PP)					SEM^e^	*P* values^f^				
	0	25	50	75	100		M	PP	M × PP	L	Q
	Total VFA, mmol/L										
VEG	32.4^c^	35.9^b^	33.5^bc^	33.7^bc^	40.0^a^	1.44	< 0.001	0.001	< 0.001	< 0.001	0.020
FL	28.1	24.4	26.0	26.5	27.5					0.306	0.215
SP	26.7^a^	26.2^a^	23.9^b^	23.1^bc^	21.9^c^					< 0.001	0.723
	Acetate (A), % of the total VFA										
VEG	59.4^c^	60.6^c^	62.4^b^	63.0^b^	65.4^a^	0.63	0.004	< 0.001	0.001	< 0.001	0.569
FL	58.9^e^	61.3^d^	65.2^c^	67.3^b^	69.8^a^					< 0.001	0.457
SP	59.2^c^	60.7^c^	64.7^b^	65.1^b^	67.7^a^					< 0.001	0.601
	Propionate (P), % of the total VFA										
VEG	32.0^a^	29.3^b^	28.0^bc^	26.9^c^	24.2^d^	0.43	0.122	< 0.001	0.008	< 0.001	0.954
FL	33.5^a^	30.1^b^	26.7^c^	25.2^d^	23.3^e^					< 0.001	0.001
SP	32.6^a^	30.5^b^	27.8^c^	26.4^d^	24.3^e^					< 0.001	0.258
	Butyrate, % of the total VFA										
VEG	7.6^b^	8.4^ab^	8.3^ab^	8.4^ab^	8.5^a^	0.57	< 0.001	< 0.001	< 0.001	0.096	0.261
FL	7.4^ab^	7.8^a^	6.9^b^	5.9^c^	5.3^c^					< 0.001	0.073
SP	7.8^a^	7.9^a^	6.8^b^	7.3^ab^	6.5^b^					0.006	0.872
	Branch-chained VFA, % of the total VFA										
VEG	1.1^c^	1.6^ab^	1.3^bc^	1.7^ab^	1.9^a^	0.14	< 0.001	< 0.001	0.284	0.006	0.907
FL	0.3^c^	0.9^b^	1.2^b^	1.5^a^	1.6^a^					< 0.001	0.015
SP	0.4^c^	0.9^b^	0.9^b^	1.3^a^	1.5^a^					< 0.001	0.603
	A:P ratio										
VEG	1.87^d^	2.08^c^	2.24^bc^	2.36^b^	2.72^a^	0.057	0.102	< 0.001	< 0.001	< 0.001	0.236
FL	1.76^e^	2.04^d^	2.44^c^	2.70^b^	3.00^a^					< 0.001	0.567
SP	1.82^e^	1.99^d^	2.33^c^	2.47^b^	2.81^a^					< 0.001	0.397

^a,b,c,d^Means with different superscript letters in the same row differ (*P* < 0.05)

^e^SEM: standard error of the means

^f^*L* linear, *Q* quadratic, These are linear and quadratic contrasts across PPC percentages within PPC maturity

**Fig. 2:**
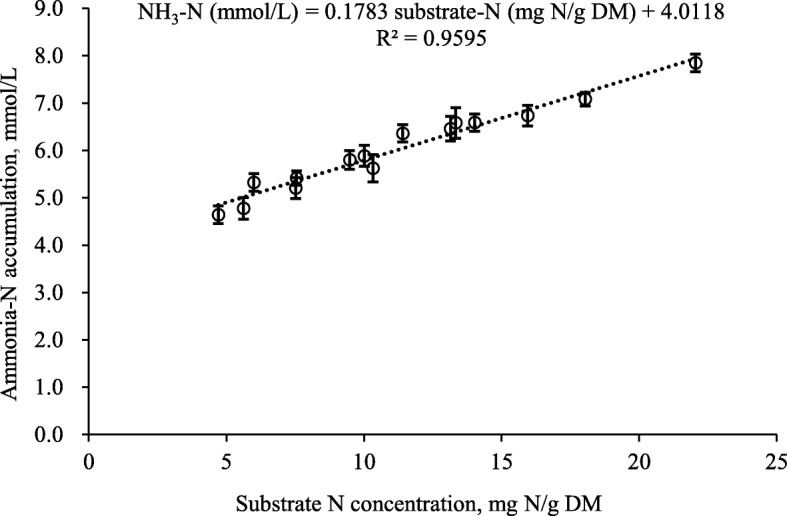
Relationship between ammonia-N accumulation at 48-h *in vitro* incubation and substrate N concentration of mixtures of purple prairie clover (PPC; *Dalea purpurea* Vent.) and cool-season grasses that were harvested when PPC reached the vegetative, full flower and seedpod stages

Ammonia-N accumulation after 48-h incubation was affected by maturity (*P* < 0.05) and proportion (*P* < 0.001) of PPC. Regardless of PPC proportion, incubation of plants at the VEG stage resulted in higher (*P* < 0.05) ammonia-N accumulation than either FL or SP stages. Increasing PPC in the mixtures linearly increased (*P* < 0.001) ammonia-N accumulation at all maturities. Regression showed that irrespective of PPC maturity, there was a linear increase (*P* < 0.01) in *in vitro* ammonia-N with increased N content of the substrate as the result of increasing levels of PPC in the mixtures (Fig. 2).

The effects of plant maturity and proportion of PPC in the mixture and their interaction on total VFA production were consistent with their effects on GP and IVDMD (Table 3). As PPC increased, total VFA production linearly increased (*P* < 0.001) at the VEG stage, but linearly decreased (*P* < 0.001) at the SP stage. For all three maturities, increasing PPC linearly increased (*P* < 0.001) molar percentage of acetate and branched-chain VFA, but linearly decreased (*P* < 0.001) molar percentage of propionate, resulting in a linear increase (*P* < 0.001) in the acetate: propionate ratio. With plants harvested at FL and SP stages, molar percentage of butyrate linearly decreased (*P* < 0.01) as PPC in the mixture increased.

### *In situ* ruminal degradation characteristics of PPC and grass mixtures at different maturities

**Fig. 3:**
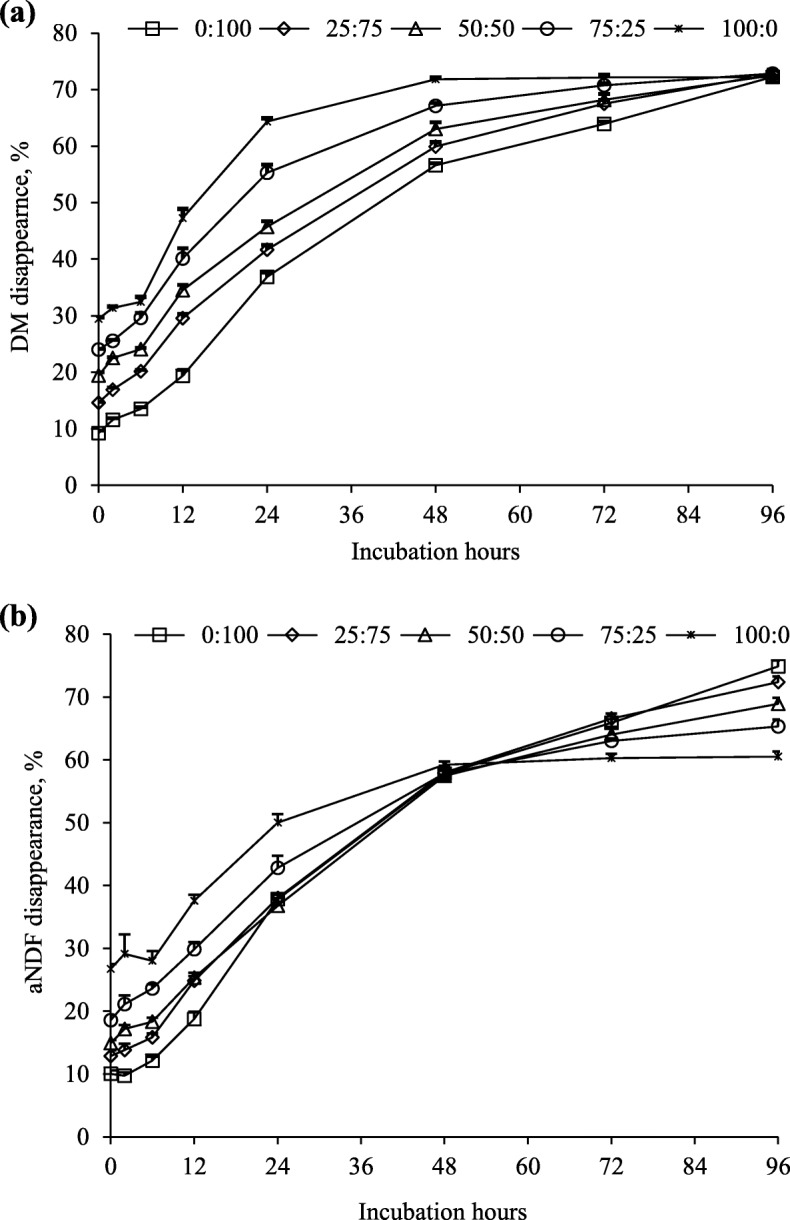
Disappearances of dry matter (DM, **a**) and neutral detergent fibre (aNDF, **b**) during 96-h incubation of mixtures of purple prairie clover (PPC; *Dalea purpurea* Vent.) and cool-season grasses in ratios of 0:100, 25:75, 50:50, 75:25 and 100:0. Both PPC and grass were harvested when PPC reached full flower stage

**Fig. 4:**
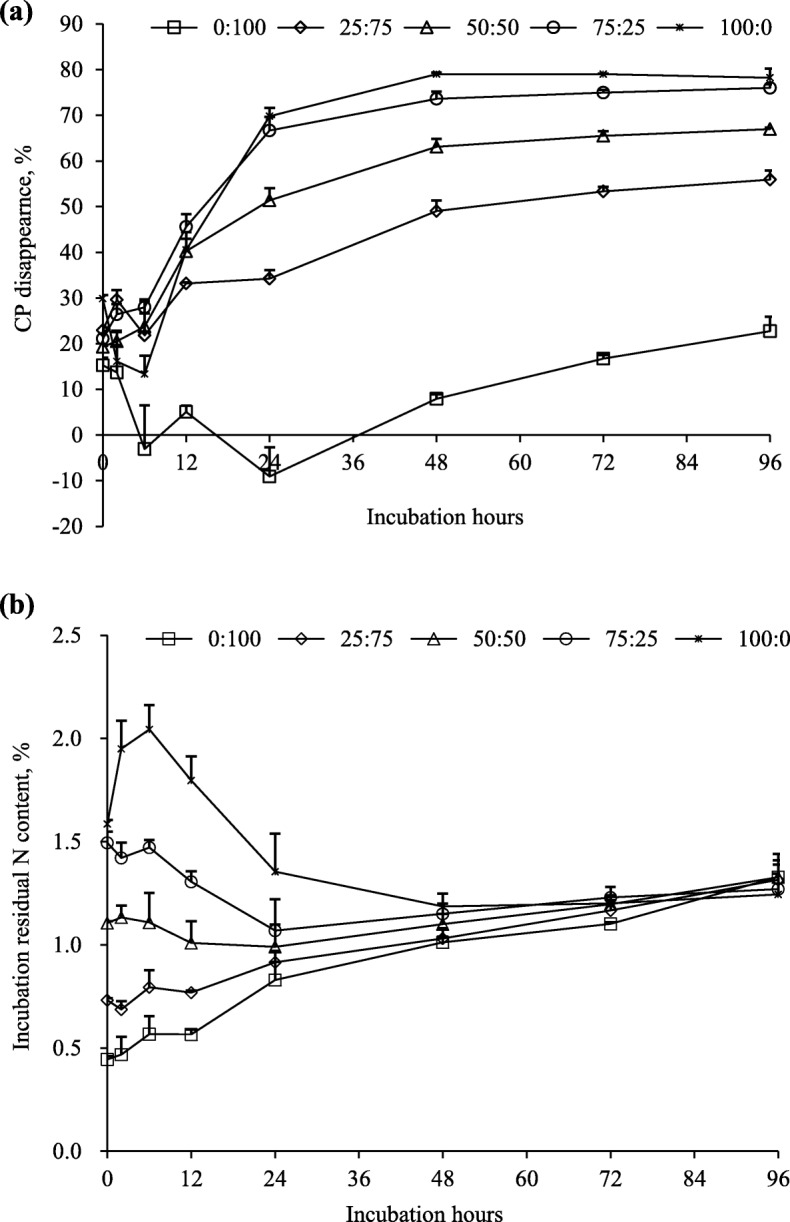
Disappearance of crude protein (CP, **a**) and residual N content (**b**) during 96-h incubation of mixtures of purple prairie clover (PPC; *Dalea purpurea* Vent.) and cool-season grasses in ratios of 0:100, 25:75, 50:50, 75:25 and 100:0. Both PPC and grasses were harvested when PPC reached the full flower stage

Dry matter disappearance linearly increased (*P* < 0.001) as PPC increased with difference being significant (*P* < 0.01) up to 72 h of ruminal incubation (Fig. 3a). However, all substrates exhibited similar DM disappearance after 96 h of incubation. The disappearance of aNDF in all substrates at 0, 2, 6 and 12 h followed the similar trend to DM disappearance (Fig. 3b). In contrast, after 72 h, aNDF disappearance linearly decreased (*P* < 0.001) as PPC increased and the difference among substrates was significant (*P* < 0.001) after 96 h of incubation. The ranking of disappearance of CP among different substrates differed at the early hours (2, 12 and 24 h) of incubation (Fig. 4a). However, disappearance of CP after 24 h linearly increased (*P* < 0.001) with increasing PPC. The change of N content of the residues over 96-h ruminal incubation differed among substrates (Fig. 4b). However, all substrates had similar residual N content at 96 h of incubation.

Increasing FL PPC in the mixture linearly and quadratically (*P* < 0.01) increased *a* fraction of DM and aNDF, but linearly and quadratically decreased (*P* < 0.01) the *b* fraction, resulting in a linear (*P* < 0.05) and quadratic (*P* < 0.01) decrease in DM and aNDF degradability (*a* + *b*, Table 4). However, the degradation rate (*c*) of the *b* fraction was linearly and quadratically increased (*P* < 0.01) for both DM and aNDF with increasing PPC. Effective degradabilities of DM and aNDF were linearly and quadratically increased (*P* < 0.05), and CP was quadratically increased (*P* < 0.05) with increasing FL PPC, with the greatest effective degradability being observed with PPC:grass ratios between 50:50 and 75:25.

**Table 4:** *In situ* ruminal degradation characteristics of dry matter (DM), neutral detergent fiber (aNDF), and crude protein (CP) disappearance of mixtures of purple prairie clover (PPC; *Dalea purpurea* Vent.) and cool-season grasses in ratios of 0:100, 25:75, 50:50, 75:25 and 100:0. Both PPC and grass were harvested when PPC reached the full flower stage

Items^g^	0:100	25:75	50:50	75:25	100:0	SEM^e^	*P* values^f^		
							*P*	L	Q
DM									
*a + b*	75.0^ab^	79.3^a^	77.6^a^	74.8^ab^	72.5^b^	1.20	0.009	0.029	0.01
*a*	10.4^e^	14.3^d^	18.3^c^	21.8^b^	30.5^a^	0.39	< 0.001	< 0.001	< 0.001
*b*	64.6^ab^	65.0^a^	59.3^b^	53.0^c^	42.0^d^	1.27	< 0.001	< 0.001	< 0.001
*c*	2.79^b^	2.46^b^	2.65^b^	3.83^b^	9.14^a^	0.476	< 0.001	< 0.001	< 0.001
*Lag*	5.1^a^	1.2^b^	0.0^b^	0.0^b^	6.0^a^	0.62	< 0.001	< 0.001	0.757
*ED*_0.05_	25.9^d^	34.0^c^	38.8^b^	44.6^a^	42.0^ab^	0.86	< 0.001	< 0.001	< 0.001
aNDF									
*a + b*	79.9^a^	80.2^a^	80.8^a^	70.7^b^	61.6^c^	1.15	< 0.001	< 0.001	< 0.001
*a*	10.1^c^	12.4^bc^	13.2^bc^	17.2^b^	26.5^a^	1.23	< 0.001	< 0.001	0.008
*b*	69.8^a^	67.8^a^	67.6^a^	53.4^b^	35.1^c^	2.15	< 0.001	< 0.001	< 0.001
*c*	2.69^b^	2.38^b^	1.93^b^	2.71^b^	5.59^a^	0.384	< 0.001	< 0.001	< 0.001
*Lag*	6.2	3.2	0.0	0.0	4.7	1.49	0.052	0.217	0.008
*ED*_0.02_	40.1^b^	44.3^ab^	46.4^ab^	47.8^a^	45.3^ab^	1.57	0.049	0.018	0.038
CP									
*a + b*	NA	67.0^b^	68.2^b^	75.8^ab^	78.2^a^	2.21	0.015	0.003	0.724
*a*	NA	23.5	16.4	22.0	19.9	2.42	0.158	0.656	0.339
*b*	NA	43.5	51.8	53.8	58.3	3.58	0.089	0.018	0.656
*c*	NA	1.65^c^	4.65^bc^	8.67^b^	13.69^a^	1.00	< 0.001	0.007	0.456
*Lag*	NA	0^b^	0^b^	4.9^ab^	8.6^a^	1.45	0.008	0.002	0.245
*ED*_0.05_	NA	33.9	41.0	41.9	29.1	3.20	0.072	0.411	0.015

^a,b,c,d^Means with different lowercased letters in the same row differ (*P* < 0.05)

^e^*SEM* standard error of the means

^f^*P* values are shown for *P* overall, *L* linear effect, *Q* quadratic effect

^g^*a*: soluble fraction (%); *b*: potentially degradable fraction (%); *a* + *b*: the maximum potential degradability; *c*: the rate at which *b* is degraded (%/h); ED_xxx_: effective degradability (%) at a ruminal passage rate of 0.05/h for DM, and 0.02/h for aNDF, and 0.05/h for CP; NA: value not be estimated

**Fig. 5:**
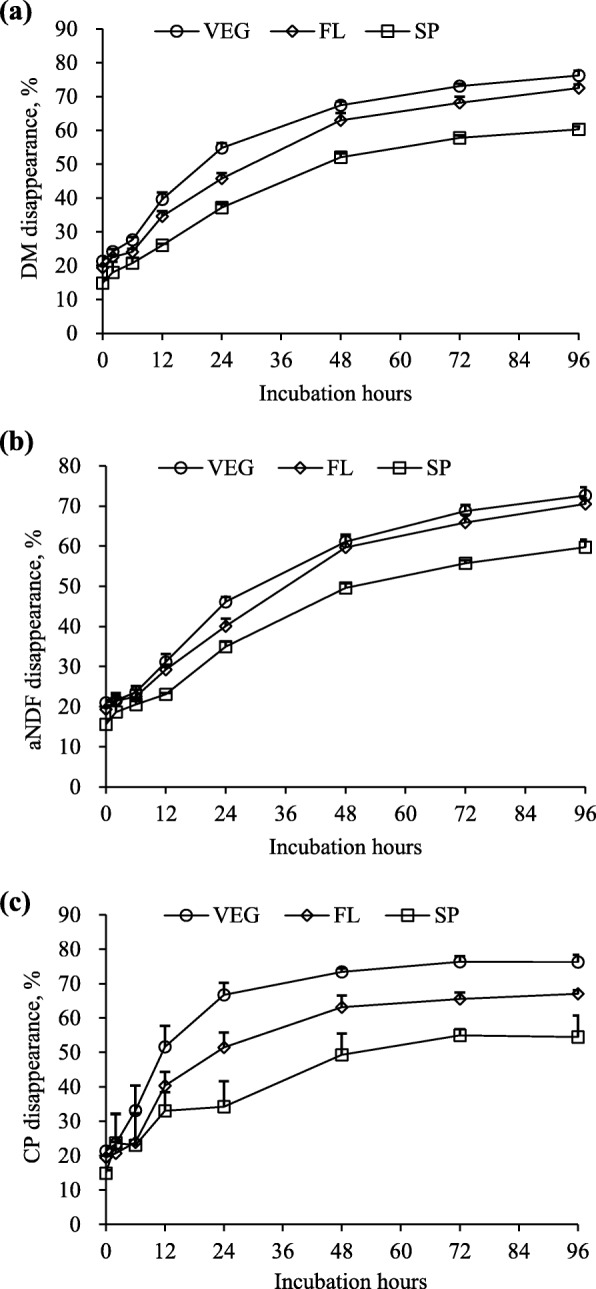
Disappearances of dry matter (DM, **a**) neutral detergent fibre (aNDF, **b**) and crude protein (CP, **c**) during 96-h incubation of mixtures of purple prairie clover (PPC; *Dalea purpurea* Vent.) and cool-season grasses in the ratio of 50:50. Both PPC and grass were harvested when PPC reached the vegetative (VEG), full flower (FL) and seedpod (SP) stages

Ruminal degradabilities of DM, aNDF and CP decreased (*P* < 0.01) as the PPC matured (Fig. 5a, b, c and Table 5). The decreases in DM and aNDF degradability were mainly observed between the FL to SP stage. However, CP degradability decreased to a similar extent with advancing maturity.

**Table 5:** *In situ* ruminal degradation characteristics of dry matter (DM), neutral detergent fiber (aNDF), and crude protein (CP) disappearance of a 50:50 mixture of purple prairie clover (PPC; *Dalea purpurea* Vent.) and cool-season grasses. Both PPC and grass were harvested when PPC reached the vegetative (VEG), full flower (FL) and seedpod (SP) stages

Items^d^	VEG	FL	SP	SEM^c^	*P-*value
DM					
*a + b*	78.3^a^	77.6^a^	66.3^b^	0.80	< 0.001
*a*	19.8^a^	18.3^a^	14.4^b^	0.74	0.002
*b*	58.5^a^	59.3^a^	51.9^b^	0.67	< 0.001
*c*	3.50^a^	2.65^b^	2.46^b^	0.081	< 0.001
*Lag*	0	0	0	–	–
*ED*_0.05_	43.9^a^	38.8^b^	31.5^c^	0.45	< 0.001
aNDF					
*a + b*	77.7^a^	80.8^a^	60.0^b^	2.20	0.001
*a*	15.3^a^	13.2^a^	7.5^b^	0.75	< 0.001
*b*	62.3^a^	67.6^a^	52.6^b^	2.43	0.021
*c*	2.64	1.93	2.71	0.274	0.156
*Lag*	1.9^ab^	0.0^b^	5.9^a^	1.20	0.034
*ED*_0.02_	47.0^a^	46.4^a^	30.3^b^	1.59	0.002
CP					
*a + b*	76.9^a^	68.2^ab^	59.7^b^	3.70	0.058
*a*	18.0	16.4	18.4	2.24	0.788
*b*	58.9^a^	51.8^ab^	41.3^b^	3.34	0.027
*c*	6.69	4.58	3.60	0.889	0.135
*Lag*	0	0	2.0	1.14	0.444
*ED*_0.05_	51.3^a^	41.0^b^	32.0^b^	3.29	0.011

^a,b^Means with different lowercased letters differ (*P* < 0.05)

^c^*SEM* standard error of the mean

^d^*a*: soluble fraction (%); *b*: potentially degradable fraction (%); *a* + *b*: the maximum potential degradability; *c*: the rate at which *b* is degraded (%/h); ED_xxx_: effective degradability (%) at a ruminal passage rate of 0.05/h for DM, and 0.02/h for NDF, and 0.05/h for CP

Degradation kinetic parameters showed similar trends. Forages harvested at VEG and FL had similar *a*, *b* and *a* + *b* fractions of DM and aNDF, and both were greater (*P* < 0.05) than that harvested at the SP stage (Table 5). Effective degradability of DM ranked as VEG > FL > SP (*P* < 0.001), whereas the ED of aNDF was similar between VEG and FL and both were greater (*P* < 0.01) than SP. Forages harvested at VEG, FL and SP had similar soluble fraction (*a*) of protein, but the potential degradable fraction (*b*) of protein was greater (*P* < 0.01) for forage harvested at the VEG than the SP stage.

## Discussion

The higher aNDF and ADF contents but lower CP content of cool-season grasses as compared to PPC across all maturities indicated that nutritional quality of PPC was superior to mature cool-season grasses. Therefore, incorporation of PPC into cool-season grass pasture would increase the forage quality of rehabilitated native pasture. However, the increased aNDF and ADF but decreased CP content of PPC with advancing maturity indicates that the nutritive value of PPC decreased as it matured. This compared with the observation that cool-season grasses had relatively similar aNDF, ADF and CP contents during the same period of growth indicated that the PPC and cool-season grass mixture had a higher nutritive value when PPC was in the vegetative stage. The nutrient composition of PPC at VEG and FL were comparable to that reported by Jin et al. [11]. Generally, with advancing maturity, forage quality declines as fibre content increases and protein concentration decreases [27]. The increased fibre content in whole plant PPC with advancing maturity was likely due to increased fibre concentration in stem and an increase in the stem: leaf ratio [11]. Reduced protein content in PPC as the plant matures was also reported by Posler et al. [28]. Nevertheless, the higher protein concentration of PPC throughout the growing season as compared to cool-season grasses suggests that PPC could be a valuable N source in rehabilitated native pasture. Others have concluded that PPC in the vegetative and flower stages are good quality forages for ruminants [29] as the fibre and CP contents are similar to other common legumes such as alfalfa and sainfoin harvested at the same growth stages [30].

Changes in CT concentration in PPC with advancing maturity were similar to that reported by Jin et al. [11]. The finding that the ECT in PPC was higher in FL but lower in SP as compared to VEG stage was also consistent with the observations of Li et al. [31]. This was mainly attributable to the higher proportion of flowers in FL and more stems in SP than VEG [11, 30, 32]. As PPC matures, the ECT concentration decreases in stem, but remains relatively constant in the leaves and flowers [32].

Gas and VFA are major products of microbial fermentation of diets in the rumen and therefore the changes of gas and VFA production due to plant maturity and PPC proportions in the forage mixtures were positively related to IVDMD in this study. The *in vitro* study showed that PPC at VEG stage had greater DM digestibility, resulting in greater GP and total VFA production than cool-season grasses. However, as PPC matured to the FL and SP stages, IVDMD, GP and total VFA production were dramatically decreased, a response that was less apparent for cool-season grasses. This combined with the same trend of nutrient composition changes of the two forages from VEG to SP indicated that digestible DM of PPC at VEG stage was higher than that of cool-season grasses, whereas the reverse occurred when PPC reached the SP stage because of the faster decline in digestible DM during transition from VEG to SP stage. These differential changes of digestible DM content between the two forages as the plant maturity advanced impacted the nutritive values of their mixtures at different maturities, resulting in plant maturity × PPC proportion interaction. However, it needs to be pointed out that in real field conditions it is not always possible to attain the desirable ratio between legume and grasses and to synchronize their stages of maturity at the time of cutting. The IVDMD, GP and total VFA production demonstrated that increasing VEG PPC in the mixtures linearly increased ruminal fermentation, but it was decreased by increasing SP PPC. In contrast, the relatively similar IVDMD, GP and total VFA of FL PPC-grass mixtures indicated that PPC and cool-season grasses were fermented to a similar degree at this stage. The decline in ruminal digestion of PPC and cool-season grasses from VEG to SP was supported by the *in situ* results which found that the ruminal degradabilities of DM, aNDF and CP in 50:50 PPC-grass mixture all declined with advancing PPC maturity. Others have also observed reduced ruminal degradation of DM and CP with advancing PPC maturity [11, 28]. Altogether, the *in vitro* ruminal digestion and productions of total VFA and gas suggest that PPC is likely to result in the greatest improvement in mixed forages when it is at the vegetative and full flower stages.

The reduction in the ruminal fermentation of PPC and cool-season grasses from vegetative to seedpod stages was reflected in the increase in ADF content of the two forages. Negative effect of ADF content on forage digestion has been demonstrated in literature [33]. Interestingly, almost all measurements in the *in vitro* experiment in this study showed linear but not quadratic responses to the proportions of PPC in the mixtures. This indicated that there was no associative effect of combining PPC and cool-season grasses on the nutritive value as observed in legume [red clover (*Trifolium pratense* L.) and white clover (*Trifolium repens* L.)]-grass (timothy (*Phleum pratense* L.) and smooth meadow grass (*Poa pratensis* L.) mixed forages [34]. A quadratic response in DM, NDF and CP effective degradabilities to increasing PPC was observed with them plateauing when FL PPC was included in the mixture at 75%. However, there were not statistical differences between 75% PPC mixture and pure PPC or other PPC-grass mixtures (aNDF and CP). This also suggested that there was no positive associative effect of mixing cool-season grass and PPC on DM, aNDF and CP effective degradability when full flower PPC was mixed with cool-season grasses. This phenomenon was largely associated with the lower ADF and higher CP contents in the full flower PPC than in cool-season grass. Dal Pizzol et al. [7] reported positive associative effects on *in vitro* fermentation as a result of mixing a tropical grass (axonopus, *Axonopus catharinenses*) and a temperate legume (alfalfa, *Medicago sativa*) but not between peanut vines (*Arachis pintoi*), sainfoin (*Onobrychis viciifolia*) and grasses of axonopus and tall fescue (*Festuca arundinacea*).

Crude protein content in PPC was higher than that in cool-season grasses throughout the growing season. This resulted in an increase in the protein concentrations of the PPC-grass mixtures and the subsequent increase in ammonia-N concentration during *in vitro* fermentation. Ammonia-N accumulation in closed *in vitro* system is the net result of ammonia from the degradation of dietary protein and utilization by microbes for microbial protein synthesis. The linearly-increased ammonia-N accumulation with increasing PPC at all maturities is a reflection of the increasing protein concentration in the mixture. Whether microbial protein synthesis (ammonia-N utilization) was affected by the inclusion of PPC was not determined in this study. Nevertheless, Jin et al. [3] found that incorporation of PPC at full flower/early seedpod stage into cool-season grasses up to 44.8% linearly increased microbial protein synthesis. One of the most common effects of dietary CT on protein degradation in the rumen is a decrease in ruminal ammonia concentrations [35]. Decreased ammonia production by PPC CT was also reported in our previous studies [10, 11, 13]. In this study, regardless of plant maturity, PPC contained higher amounts of protein and CT and produced more ammonia-N than cool-season grasses. The linearly-increased protein degradability of the PPC-grass mixtures as the protein concentration increased was consistent with the increase in ammonia-N production in the *in vitro* experiment. Dal Pizzol et al. [7] also reported that incorporation of legume forage (sainfoin) containing CT into grass linearly increased ruminal ammonia-N production. The decrease of protein disappearance of PPC: grasses mixture at the ratio of 0:100 on 2, 12, and 24 h of the incubation was due to the increased microbial colonization that surpassed the protein disappearance from the feed particles during this period of incubation. This phenomenon is commonly observed with poor quality roughages of low N content [36, 37]. Because DM and CP disappearances were not corrected by microbial N and microbial mass, these values were underestimations of the corresponding true DM and CP disappearances in this study. In addition, Figs. 3 and 4 showed that disappearances of DM, aNDF and CP slightly increased for mixture with high proportion of grasses (100% and 75%) between 72 and 96 h of the ruminal incubation. Therefore, there might be a chance that the ruminal degradation of these substrates did not reached a plateau at the 96-h incubation, which might slightly affect the kinetic parameters estimated from them. Ruminal incubation longer than 96-h and correction for microbial N contamination in the incubation residue are needed to accurately estimate the ruminal degradation parameters for such feed types.

The result that increasing PPC proportion in the PPC-grass mixture increased acetate: propionate ratio by increasing acetate and decreasing propionate was consistent with Jin et al. [3]. The variations of these major VFAs caused by the inclusion of PPC may be specific to the two types of the forages used in this study, as Burke et al. [38] compared the VFA profiles of eight temperate grasses and six temperate legumes and found no difference in VFA profiles after ruminal fermentation of these various species. It is also likely that the antimicrobial activity of CT in PPC contributed partially to the variation in VFA profiles. The effects of CT in PPC on increasing acetate: propionate ratio during ruminal fermentation has been demonstrated by both *in vitro* and *in vivo* studies [3, 11, 13]. The negative effect of PPC CT on propionate production might reflect the ability of these phenolics to inhibit specific members of the microbiota, such as *Prevotella bryantii* [39] or *Ruminobacter amylophilus* [40], because both of these produce propionate in the rumen [41].

It is generally regarded that the nutritional role of CT in ruminant nutrition depends on their dietary concentrations and chemical composition [11, 42, 43]. Purple prairie clover was the only forage that contained CT in this study and the CT concentration increased as increasing PPC was added to the grass mixture. Both *in vitro* and *in situ* studies showed PPC CT at concentrations up to 82 g/kg DM had minimal impact on ruminal feed digestion [11, 12]. In contrast, CT in other temperate forages have been shown to have negative effects on nutrient digestion at CT concentrations > 50 g/kg DM [35]. Huang et al. [32] found that PPC CT were predominantly composed of procyanidins with less —OH than prodelphinidins type and as a result lower biological activity. The same authors also found that reactivity of PPC CT with proteins decreased with advancing PPC maturity as a result of increased mean degree of polymerization. Therefore, the reduction in ruminal digestion with advancing maturity in PPC is likely a result of increased ADF deposition rather than as a result of the presence of CT.

## Conclusion

Purple prairie clover contained higher protein than cool-season grasses throughout the growing season and therefore the incorporation of PPC into cool-season grasses would increase the protein content of forage in rehabilitated native pasture. However, the improvement in nutritive value of the forage by the incorporation of PPC into native pasture depends on the PPC growth stage, with greatest benefit being obtained at the vegetative stage followed by full flower and seedpod stages. Considering the faster decline of nutrient digestion of PPC over the growth season than cool-season grasses and balanced by N content of the two types of forages, it seems that about 50% of PPC in the PPC-grass mixed forage would provide most benefit for the purpose of extending the grazing season. These results need to be confirmed with field trials to better understand competition ability between PPC and cool season-grasses.

## Data Availability

The datasets supporting the conclusions of this article are included within the article.

## Abbreviations

A: Acetate
ADF: Acid detergent fibre
aNDF: neutral detergent fibre
CP: Crude protein
CT: Condensed tannins
DM: Dry matter
ECT: Extractable condensed tannins
ED: Effective degradability
FL: Full flower
GP: Gas production
IVDMD: *In vitro* dry matter disappearance
LRDC: Lethbridge Research and Development Centre
N: Nitrogen
OM: Organic matter
P: Propionate
PPC: Purple prairie clover
SP: Seedpod
VEG: Vegetative
VFA: Volatile fatty acid
